# Silver nanoparticles at sub-cytotoxic levels increase enteric pathogen invasion by compromising intestinal epithelial barrier integrity

**DOI:** 10.3389/fcimb.2026.1745955

**Published:** 2026-02-17

**Authors:** Radwa A. Hanafy, Kuppan Gokulan, Sangeeta Khare

**Affiliations:** Division of Microbiology, National Center for Toxicological Research, US Food and Drug Administration, AR, Jefferson, United States

**Keywords:** AgNP, enteric pathogen, gastrointestinal tract, invasion, oral exposure

## Abstract

**Introduction:**

Silver nanoparticles (AgNPs) are increasingly used due to their antimicrobial properties and incorporated into food packaging and dietary supplements. However, their potential to disrupt intestinal epithelial integrity and enhance susceptibility to bacterial infection remains insufficiently characterized. Building on our previous *in vivo* and *in vitro* findings that AgNP exposure alters epithelial gene expression, cytokine secretion, and gut microbial composition, this study evaluated how AgNPs affect host–pathogen interactions at the intestinal barrier.

**Methods:**

This study aimed to examine the effect of 10 nm AgNPs pretreatment to human intestinal epithelial cells (T84 cells) at the sub-cytotoxic concentrations to determine adherence, invasion and intracellular persistence of *Salmonella* serovar Heidelberg, a frequent cause of foodborne outbreaks in North America and Europe. Epithelial barrier permeability, gene expression profiles, and cytokine responses were also assessed following AgNP exposure and bacterial infection.

**Results:**

Pretreatment of intestinal epithelial cells with AgNPs (10 or 20 µg/mL) did not affect bacterial initial adhesion. However, 10 µg/mL AgNPs significantly increased bacterial invasion and intracellular persistence, demonstrating impaired epithelial defenses in a concentration-dependent manner. The preexposure to AgNP upregulated multiple intestinal permeability-associated genes that are involved in tight and gap junctions, focal adhesions, and cytoskeletal remodeling, with the 10 µg/mL concentration and bacterial infection showing the most statistically significant changes. This response suggests a compensatory repair mechanism to maintain barrier integrity, however it was insufficient to prevent pathogen invasion and intracellular survival. Additionally, 10 µg/mL AgNPs pretreatment significantly increased the secretion of proinflammatory cytokines (e.g., IL-18, TNF-α), while decreased the level of important epithelial repair and anti-inflammatory cytokines (e.g., G-CSF, IL-1ra). These results suggest that exposure to AgNPs, even at low concentration, can impair intestinal barrier integrity, hence facilitating pathogen invasion and persistence.

**Discussion:**

Given the increasing use of AgNPs into orally ingested consumer products, these results underscore a potential health risk associated with chronic AgNPs ingestion and highlights the need to re-evaluate their safety in the context of gastrointestinal health and host-pathogen interactions.

## 1 Introduction

Silver nanoparticles (AgNPs) are widely used as antimicrobial agents in many consumer products, including textiles, medical devices, personal care products, cosmetics, food storage containers, and packaging materials (Dos Santos et al., 2014; Lohani et al., 2014; Tulve et al., 2015; Joshi et al., 2021; Kaiser et al., 2023). Due to their extensive and prolonged use, there is a growing concern about potential human exposure, particularly via ingestion, either unintentionally through leaching into foods or intentionally through the consumption of colloidal silver supplements (Ferdous and Nemmar, 2020; Rogers et al., 2020; Recordati et al., 2021). AgNPs’ exposure routes include oral ingestion, dermal contact, intravenous, or inhalation, with each route leading to the distribution of AgNPs to different tissues and organs (Kumar and Goia, 2020; Sánchez-López et al., 2020; Xu et al., 2020; Chen et al., 2024). The long-term usage of colloidal silver products can lead to argyria, a condition where skin, eyes, and nail beds are permanently discolored to blue-gray (Kwon et al., 2009; Thompson et al., 2009). Furthermore, AgNP use in dental disease management is increasing (Butler et al., 2023; Joseph et al., 2025; Yin et al., 2025). AgNPs exposure has been reported to interfere with drug absorption and leads to cytotoxic effects on kidneys, liver, and nervous system (Kwon et al., 2009; Liao et al., 2019; Olugbodi et al., 2023).

Multiple pharmacokinetic and retention studies show that AgNPs can accumulate and persist in human body for extended durations, depending on particle size, dose, and route of exposure (Cong et al., 2014; Jo et al., 2020; Li and Cummins, 2020). Consumption in the U.S. has increased despite the U.S. Food and Drug Administration (FDA) ruling that all over the counter (OTC) drug products containing colloidal silver ingredients or silver salts are not generally recognized as safe (GRAS) or effective for treating any disease or condition (FR Doc. 99–21253) (Department of Health and Human Services (HHS) and Public Health Service (PHS), Food and Drug Administration (FDA), 1999). The silver concentration and particle size in these commercial colloidal products vary widely, with concentration ranging from 0.002 to 16.4 ppm and sizes ranging from 1 nm to more than 200 nm (Kumar and Goia, 2020). This inconsistency raises more concerns about their efficacy and safety. These safety concerns have drawn significant attention to the potential health risk of AgNPs exposure, particularly its impact on the gastrointestinal tract (GIT), the major site of AgNPs exposure following oral intake (Williams et al., 2016; Orr et al., 2019; Gillois et al., 2021). The GIT serves as a critical interface between the external environment and the host, functioning not only in nutrient absorption but also as a selective barrier that limits the translocation of microbes, toxins, and foreign particles. Disruption of this epithelial barrier can increase susceptibility to infection and inflammation, making it a key target for assessing the effects of ingested nanoparticles such as AgNPs. Under normal physiological conditions, the intestinal epithelial cells are sealed by specialized intercellular junctions such as tight and gap junctions, adherens, and desmosomes. These structures are essential for maintaining the membrane integrity and selective permeability by allowing nutrients absorption and preventing the entry of harmful microorganisms and xenobiotics (Tsukita et al., 2001; Hartsock and Nelson, 2008; Groschwitz and Hogan, 2009; Chelakkot et al., 2018). Increased permeability of the intestinal barrier can allow crossing exogenous molecules and microbes triggering immune activation and infection (Vancamelbeke and Vermeire, 2017; Dmytriv et al., 2024). Disruption of the intestinal barrier, often referred to as a “leaky gut”, is associated with gastrointestinal inflammation and diseases such as inflammatory bowel disease (IBD), celiac disease, irritable bowel syndrome (IBS), diabetes, arthritis, and neuropsychiatric disorders (Bischoff et al., 2014; Mu et al., 2017; Obrenovich, 2018; Dicks et al., 2021; Boles et al., 2023; Rawat et al., 2025).

Earlier studies have demonstrated that AgNPs, especially at smaller sizes (~10 nm), can compromise gut epithelial barrier integrity (Williams et al., 2016). *In vitro* study using T84 colonic epithelial cells have demonstrated both size- and dose-dependent reductions in transepithelial electrical resistance (TEER), a measure of the intestinal barrier integrity, accompanied by altered expression of tight junction and adhesion genes and the most pronounced effects were observed at 10 nm AgNPs (Williams et al., 2016). Similarly, oral exposure to low concentrations of 10 nm AgNPs has resulted in a significant change in the gut microbial populations and alteration of mucosal immune gene expression (Williams et al., 2015; Orr et al., 2019). Additional studies have reported that AgNPs exposure reduces TEER, inhibits epithelial cell proliferation, and promotes inflammation and oxidative stress in intestinal cells (McCracken et al., 2015; Kampfer et al., 2020). Also, ex-vivo studies using human intestinal tissue confirmed that AgNPs treatment has resulted in alteration of cytokine secretion and upregulation of genes associated with increased intestinal permeability (Gokulan et al., 2020). Together, these findings suggest that even sub-cytotoxic levels of AgNPs can compromise the epithelial barrier integrity and increase the susceptibility to bacterial infection. Therefore, understanding of how AgNPs shape epithelial immune signaling and junctional integrity during bacterial infection is needed to evaluate their safety and biological impact in food and biomedical applications.

Enteric pathogenic bacteria such as *Salmonella enterica*, *Escherichia coli*, *Listeria monocytogenes*, and *Shigella* species have the ability to breach the intestinal epithelium and trigger inflammation by disrupting intercellular junctions and manipulating host signaling pathways (Tafazoli et al., 2003; Reis and Horn, 2010; Gokulan et al., 2013; JW. Immune evasion, 2023). While sublethal concentrations of AgNPs have been shown to alter bacterial gene expression, affect bacterial biofilm stability and potentially influence virulence (Yang and Alvarez, 2015; Grun et al., 2016), their effects on bacterial invasion and persistence within intestinal epithelial cells as well as the host cell response to these interactions, has not been thoroughly investigated.

This study aims to investigate the impact of AgNPs exposure, particularly at small size and sub cytotoxic concentrations, on intestinal epithelial barrier integrity and susceptibility to bacterial infection, specifically *Salmonella enterica* serovar Heidelberg strain 146, an enteric pathogen. This non-typhoidal Salmonella serovar is of clinical importance and is associated with poultry, foodborne outbreaks, invasive disease, and antimicrobial resistance (Folster et al., 2012; Gokulan et al., 2013; Antony et al., 2018; Gokulan et al., 2020). This work provides new insight into the potential effects of AgNPs exposure on intestinal barrier function and host susceptibility to enteric infection.

## 2 Materials and methods

### 2.1 T84 cell culture

Human colon carcinoma T84 epithelial cells (ATCC^®^ CCL-248™) were cultured in complete medium composed of Dulbecco’s Modified Eagle Medium/Ham’s F-12 (DMEM/F-12) supplemented with l-glutamine and HEPES (ATCC, Manassas, VA, USA), 10% fetal bovine serum (FBS), 1% penicillin-streptomycin and 0.1% fungizone (Thermo Fisher Scientific, Waltham, MA, USA). Cells were grown in complete media using 75 cm^2^ cell culture flasks until approximately 70–80% confluent and then split into 24-well plates using 0.25% Trypsin–EDTA. Cell cultures were maintained in a 37°C incubator with 5% CO2 and 95% humidity, and media were replaced every 2–3 days.

### 2.2 Bacterial strain and culture conditions

In this study, we used *Salmonella enterica* serovar Heidelberg strain 146, a multidrug-resistant isolate due to its virulence, invasiveness, and ability to persist in epithelial cells (Gokulan et al., 2013). Strain 146 contains a VirB/D4 type IV secretion system (T4SS) and IncA/C and IncI1 plasmids, responsible for its intracellular survival and antimicrobial resistance, making it a relevant model for studying epithelial barrier-pathogen interactions. Using primers and PCR protocol previously described in (Williams et al., 2017), we confirmed the presence of silver resistance genes for *silA, silE, silP* and *silS* in strain 146. For the experiments, bacterial strain was streaked onto Luria-Bertani (LB) agar and grown overnight at 37°C. A single colony was used to inoculate 5 ml LB broth media and incubated overnight at 37°C with shaking at 250 RPM. Then, 100 µL of overnight culture was inoculated into 5 ml fresh LB media with shaking at 250 RPM at 37°C until the culture reached mid log phase (OD^600^ between 0.6 to 0.8).

### 2.3 Silver nanoparticles preparation and characterization

Earlier study showed that smaller-sized nanoparticles exhibit higher cellular uptake compared to larger particles (Williams et al., 2016). We used 10 nm AgNPs at sub-cytotoxic concentration (10–20 µg/mL) to assess the AgNPs dose-dependent effects on epithelial barrier alteration and host-pathogen interactions. The selected size and concentration of AgNP does not show any corona formation (Williams et al., 2016). Well characterized citrate-stabilized AgNPs with a diameter of 10 nm were obtained from NanoComposix (San Diego, CA, USA). Detailed characterization of the AgNPs including, diameter, pH, mass concentration, silver purity, and zeta-potential, was provided by the company. Nanoparticles sterility was confirmed by plating them on LB agar and incubated at 37°C for 48 hours. An Endotoxin assay was performed to ensure endotoxin-free AgNPs, using the ToxinSensor Chromogenic LAL Endotoxin Assay Kit (GenScript, Piscataway, NJ, USA) according to the manufacturer’s protocol to confirm lack of endotoxin contamination. AgNPs stock solution was stored in the dark at 4°C to prevent any degradation. Before use, stocks were vortexed and sonicated in a water bath sonicator for 60 seconds, then diluted to final working concentrations of 10 µg/mL and 20 µg/mL.

### 2.4 Bacterial adhesion, invasion, and persistence assays

T84 cell monolayers were used to assess bacterial adhesion, invasion, and persistence in the presence or absence of 10 nm AgNPs. T84 cells were seeded in 24-well culture plates at a density of 4 x 10^5^ cells per well and incubated at 37°C, 5% CO_2_ and 95% humidity until forming a monolayer. For AgNPs exposure, cells were pretreated for 24 hours with 10 nm AgNPs at either 10 µg/mL or 20 µg/mL. Then, culture plates were centrifuged at 500 rpm for 2 minutes before incubation to allow even distribution of the AgNPs. After AgNPs pretreatment, T84 cells were washed with pre-warmed PBS and infected with *Salmonella enterica* serovar Heidelberg strain 146 at a multiplicity of infection (MOI) of 1:200 (T84 cells: bacteria). Infected plates were centrifuged at 500 rpm for 2 minutes to promote bacterial contact and incubated for 1 hour at 37°C to allow for bacterial adhesion and internalization. After infection, the infection medium was removed and cells were washed three times with PBS to remove non-adherent bacteria, then lysed with 1 mL of 1% Triton X-100 in PBS for 5 minutes with vigorous pipetting. Short lysis duration at this low concentration of Triton is sufficient to lyse T84 cells but is not expected to kill gram negative bacteria such as *S. enterica* (Liu et al., 2001; He et al., 2013; Lee et al., 2023). Cell Lysates were serially diluted (10^-^¹ to 10^-5^) in PBS, and 100 µL aliquots from each dilution (typically 10^-3^ to 10^-5^) were plated in duplicate onto LB agar. Plates were incubated overnight at 37°C, and colony-forming units (CFUs) were counted the next day to determine the total number of bacteria associated with the cells (adherent and internalized bacteria). Infected T84 cells without AgNPs pretreatment served as a control for the adhesion assay.

For the invasion assay, after the initial 1-hour infection period, cells were washed three times with PBS and incubated with 1 mL of complete medium supplemented with 50 µg/mL gentamicin (Gibco, Thermo-Fisher Scientific) for an additional 1 hour at 37°C to kill any extracellular bacteria. Cells were then washed three times with PBS and lysed with 1% Triton X-100 as described above. Serial dilutions were plated and incubated, and CFUs were counted to determine the number of intracellular bacteria. A control group for the invasion assay consists of infected cells that were not treated with AgNPs but were subjected to gentamycin for 1 hour.

For the persistence assays, the gentamicin treatment was extended to 24 hours to evaluate bacterial survival within epithelial cells even in the presence of gentamicin. Cells were again washed, lysed, and CFUs were counted from serial dilutions after the overnight incubation on LB plates. A control group for the persistence assay consists of infected cells that were not treated with AgNPs but were subjected to gentamycin for 24 hours.

Each experimental condition was performed in biological triplicates and repeated at least three times. Quantification of bacteria was defined as follows: adhesion was calculated by subtracting invasion CFUs from total CFUs prior to gentamicin treatment; invasion was defined as the number of intracellular bacteria after 1-hour gentamicin exposure; and persistence was determined by CFU counts following 24-hour gentamicin treatment. Data were expressed as mean ± standard deviation (SD), and statistical comparisons were performed using unpaired Student’s t-tests.

### 2.5 Quantitative PCR analysis of cell junction gene expression

Total RNA was isolated from T84 cells after bacterial invasion and persistence assays using Trizol-chloroform reagent (Molecular Research Center, Cincinnati, OH). RNA was treated with the TURBO DNA-free™ Kit (Life Technologies, Frederick, MD) to eliminate any genomic DNA. RNA quality and concentrations were checked using a BioTek Cytation 5 spectrophotometer (Agilent Technologies, Santa Clara, CA). Reverse transcription for cDNA synthesis from purified RNA was performed using Superscript IV VILO Kit (Fischer Scientific, Hanover Park, IL) following the manufacturer’s protocol. Expression profile of 84 genes involved in epithelial cell junctions and barrier integrity was performed using the RT² Profiler™ PCR Array: Human Cell Junction Pathway Finder (Qiagen, Germantown, MD, USA). PCR arrays were amplified using Bio-Rad CFX384 RT-PCR (Bio-Rad, Hercules, CA, USA) instrument, with an initial denaturation step at 95°C for 10 minutes, followed by 40 cycles, with each cycle consists of 15 seconds at 95°C, and 1 minute at 60°C. Three biological replicates were analyzed for each experimental group. Gene expression data were normalized to internal reference genes provided on the array (*GAPDH* and *RPLP0*) and analyzed using Qiagen’s gene globe software (https://geneglobe.qiagen.com/us/analyze). Comparative analysis was conducted between T84 cells treated with 10 µg/mL and 20 µg/mL AgNPs, with or without bacterial exposure for 1 hour (invasion) and 24 hours (persistence) and untreated T84 cells controls. Statistical significance for differentially expressed genes was determined using unpaired Student’s t-tests, and genes with *p* < 0.05 were considered significantly regulated.

### 2.6 Measurement of cytokine concentration

Culture supernatants were collected from T84 cells at the end of bacterial invasion and persistence assays, with or without pretreatment with 10 nm AgNPs at concentrations of 10 or 20 µg/mL. Then, supernatants were centrifuged at 10000 rpm for 10 minutes at 4°C to remove any remaining bacterial cells or AgNPs residues. Supernatants were then stored at −80°C until further analysis. Cytokine secretion profiles were quantified using the Bio-Plex Pro™ Human Cytokine Screening Panel, 48-Plex (Bio-Rad Laboratories, Hercules, CA, USA), according to the manufacturer’s instructions and analyzed using the Luminex FLEXMAP 3D system (acquired from Millipore Sigma, MA, USA). All samples were run in technical duplicates, and cytokine concentrations (pg/mL) were calculated from standard. Three biological replicates were analyzed per each condition. Comparative analysis was conducted across experimental groups including untreated T84 cells (control), AgNPs-treated cells (10 or 20 µg/mL), and cells infected with *Salmonella enterica* with or without AgNPs pretreatment under both invasion and persistence conditions. Statistical significance for secreted cytokines was determined using unpaired Student’s t-tests, and cytokines with *p* < 0.05 were considered significantly changed.

## 3 Results

### 3.1 Enhanced *Salmonella* invasion and persistence following AgNPs exposure in T84 cells

To evaluate how AgNPs exposure influences *Salmonella enterica* interactions with intestinal epithelial cells, we assessed bacterial adhesion, invasion, and persistence in T84 cells following 24-hour pretreatment with 10 nm AgNPs at either 10 or 20 µg/mL. Cells were then exposed to *S. enterica* serovar Heidelberg strain 146 using three infection models: adhesion assay (1-hour infection), invasion assay (1-hour infection + gentamicin) and persistence assay (24-hour infection + gentamicin). For each condition, results were compared to corresponding AgNPs-untreated infection controls: adhesion (cells + bacteria for 1 hour), invasion (cells + bacteria + gentamicin for 1 hour), and persistence (cells + bacteria + gentamicin for 24 hour).

**Figure 1:**
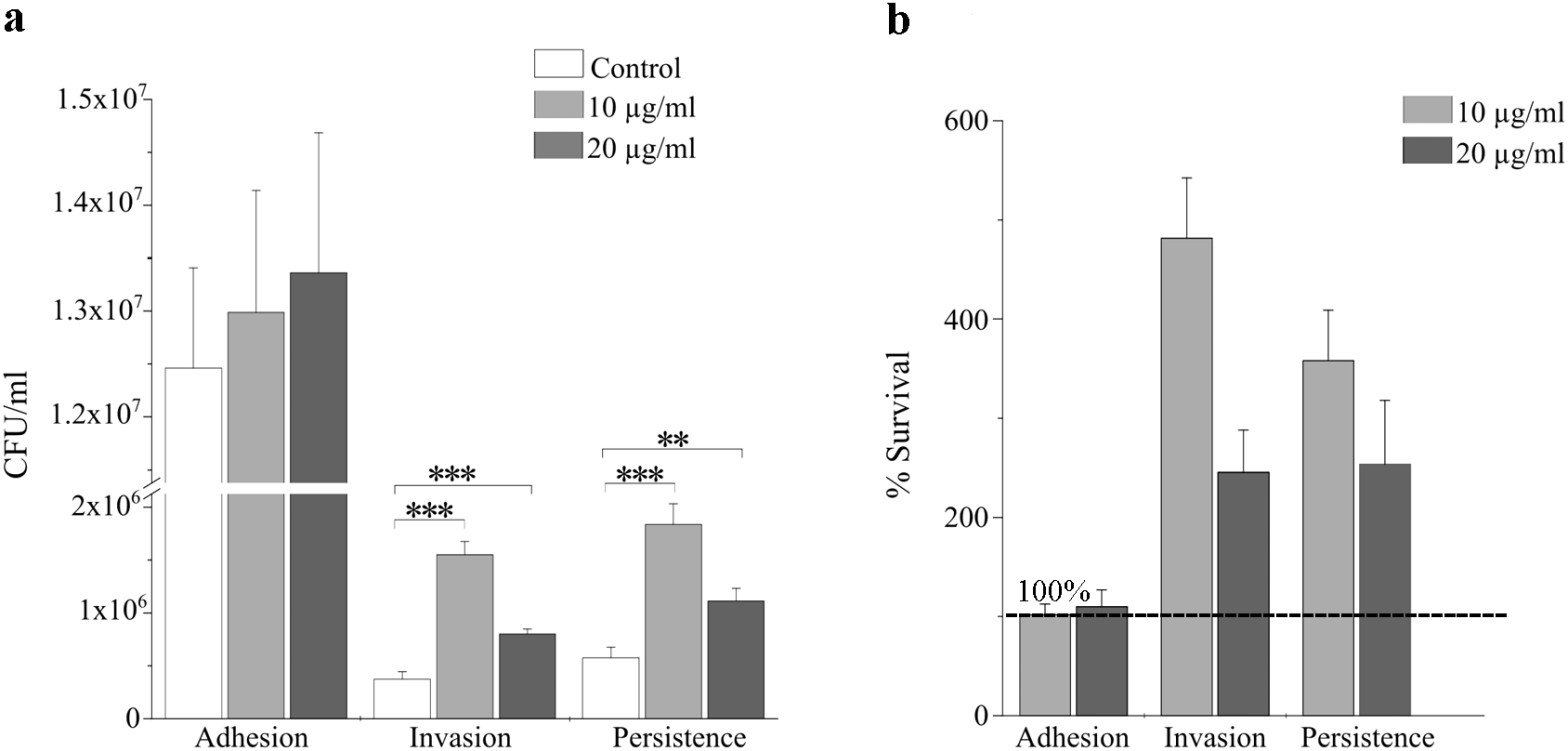
Effect of 10 and 20 µg/mL AgNPs pretreatment on bacterial adhesion, invasion, and persistence in infected T84 epithelial cells. T84 cells were pretreated or 24 hours with 10 nm AgNPs at 10 or 20 µg/mL, followed infection with *Salmonella enterica* serovar *Heidelberg* strain 146. **(a)** Bacterial adhesion, invasion, and persistence were quantified by measuring CFU/mL (as described in M&Ms). **(b)** Bacterial survival percentage in T84 cells during adhesion, invasion and persistence following AgNPs treatment. Survival was calculated by dividing CFU/mL (as described in M&Ms) for each condition by its corresponding control (set to 100% and therefore not shown as a separate bar) and expressed as a percentage. Bars represent mean ± SD from three independent experiments performed in biological triplicates. Asterisks indicate statistically significant differences compared to respective untreated infection controls (**p < 0.005, ***p < 0.0005; unpaired t-test).

AgNPs pretreatment did not significantly affect bacterial adhesion to T84 cells (**Figure 1a**). Also, counts of colony forming units (CFU) for adherent *Salmonella* remained comparable across all treatment groups, ranging from approximately 1.2 × 10^7^ to 1.3 × 10^7^ CFU/mL, suggesting that AgNPs exposure did not affect the initial attachment of *Salmonella* to the epithelial surface.

In contrast to adhesion, bacterial invasion was significantly increased following pretreatment with 10 µg/mL AgNPs compared to the invasion control, and a similar but less pronounced increase was observed at 20 µg/mL (**Figure 1a**). Similarly, bacterial persistence in the presence of gentamicin for 24 hours was significantly higher in both AgNPs-pretreated groups compared to the persistence control, with the concentration 10 µg/mL showing the most increase. Persistence assays revealed higher number of CFU/ml than those observed during the invasion (**Figure 1a**). Survival percentage data further supported these results, which showed more than a 5-fold increase in intracellular bacterial survival during invasion with 10 µg/mL AgNPs pretreatment, more than the increase observed with 20µg/mL (**Figure 1b**). Also, increased survival was observed during persistence as well, though slightly reduced at the higher AgNPs concentration (20µg/mL) (**Figure 1b**).

### 3.2 Impact of AgNPs exposure and bacterial infection on expression of human cell junction genes

Gene expression profiles were assessed using a human cell junction RT² Profiler PCR Array comprising 84 target genes. Cells were pretreated for 24 hours with 10 or 20 µg/mL of 10 nm AgNPs, either alone or in combination with bacterial infection as part of the invasion and persistence assays. Changes in gene expression were evaluated relative to untreated control T84 cells, and genes with a fold change ≥ ± 2.0 and *p* < 0.05 were considered significantly regulated. Pretreatment of T84 cells with silver AgNPs, either alone or in combination with bacterial infection, resulted in significant changes in the expression of genes involved in epithelial integrity including those associated with adherens junctions, tight junctions, gap junctions, and cell adhesion molecules.

**Figure 2:**
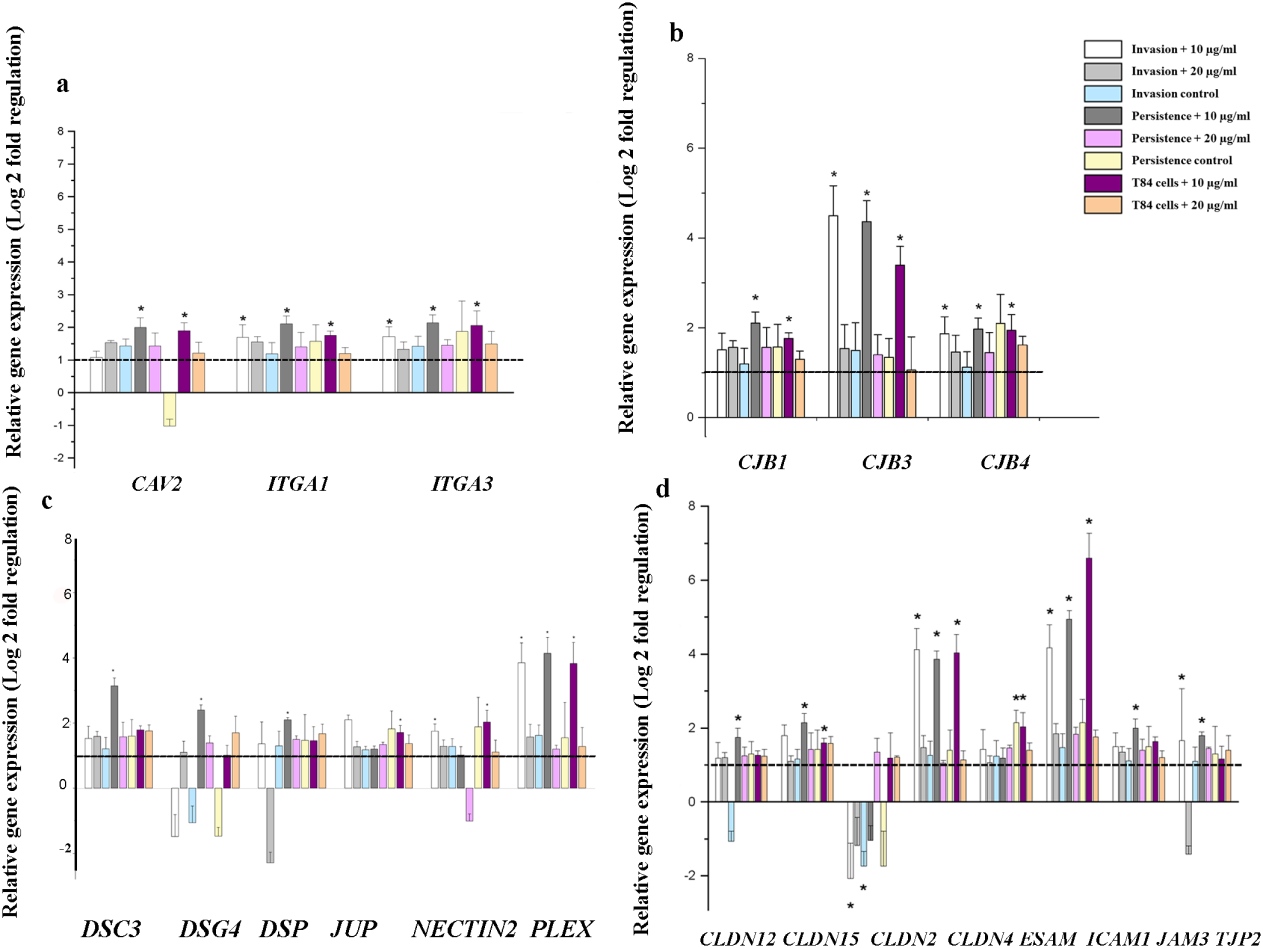
Relative expression of genes related to cell-cell junction and epithelial barrier function in T84 cells following AgNPs exposure and bacterial infection. T84 cells were pretreated with 10 nm AgNPs at either 10 µg/mL or 20 µg/mL, alone or in combination with S. enterica under invasion (1 h + gentamicin) or persistence (24 h + gentamicin) conditions. Invasion control consists of infected cells that are not pretreated with AgNPs but treated with gentamicin for 1 hour, while persistence control consists of infected cells not pretreated with AgNPs but treated with gentamicin for 24 hours. Gene expression (as described in M&Ms) was measured using RT² Profiler PCR arrays and is represented as Log_2_ fold regulation relative to untreated control cells. **(a)** Focal adhesion genes, **(b)** Gap junction genes, **(c)** Tight junction genes, and **(d)** Adherens Junctions, desmosomes, and hemidesmosomes genes. Bars represent mean ± SE from biological triplicates. Asterisks indicate statistically significant differences compared to uninfected and AgNPs untreated control (p < 0.05) (*p < 0.05, **p < 0.005; unpaired t-test).

Across all conditions, treatment with low AgNPs concentration (10 µg/mL) consistently showed the most significant changes in gene expression when compared to treatment with the higher concentration (20 µg/mL) (**Figure 2**). This pattern was apparent in both the absence and presence of bacterial infection for 1 hour (invasion) or 24 hours (persistence). The 10 µg/mL concentration led to significant gene upregulation across multiple cell junctions and permeability related genes, while the 20 µg/mL concentration had minimal and non-significant effect on the transcriptional activity. Notably, a combination of 10 µg/mL AgNPs pretreatment and bacterial infection led to significant upregulation of several key genes involved in focal adhesion (*ITGA1*, *ITGA3*, and *CAV2*) (**Figure 2a**) and gap junction (*GJB1, GJB3*, and *GJB4*), with *GJB3* showing fold changes greater than 4-fold during both invasion and persistence conditions in the presence of 10 µg/mL AgNPs (**Figure 2b**). These genes remained largely unchanged when T84 cells were infected with bacteria only, and no significant alterations were observed following treatment with 20 µg/mL AgNPs.

Similarly, bacterial infection of 10 µg/mL treated T84 cells caused strong and statistically significant changes in the expression of several tight junction-related genes (**Figure 2c**). In particular, multiple claudins, including *CLDN12, CLDN15 and CLDN4*, as well as *JAM3* and *TJP2* were significantly upregulated with fold increases ranging from approximately 2- to 4-fold, while *CLDN2* was downregulated (**Figure 2c**). *ICAM1* and *ESAM*, were upregulated, with *ICAM1* reaching fold changes exceeding 6-fold (**Figure 2c**).

Also, bacterial invasion and persistence in the presence of 10 µg/mL AgNPs resulted in upregulation of genes associated with adherens junctions, desmosomes and hemidesmosomes (**Figure 2d**). Among these, *PLEC* showed the highest and statistically significant changes in expression, exceeding a 3-fold increase compared to untreated controls. *DSP, DSC3, DSG4*, *NECTIN2*, and *JUP* were also significantly upregulated (**Figure 2d**). In contrast, 20 µg/mL AgNPs had minimal or non-significant on these genes. Bacterial infection alone led to a moderate downregulation of *DSG4* and *NECTIN2* but did not significantly change other genes in this category (**Figure 2d**).

### 3.3 AgNPs exposure alters cytokine secretion profiles in intestinal epithelial cells during bacterial infection

**Figure 3:**
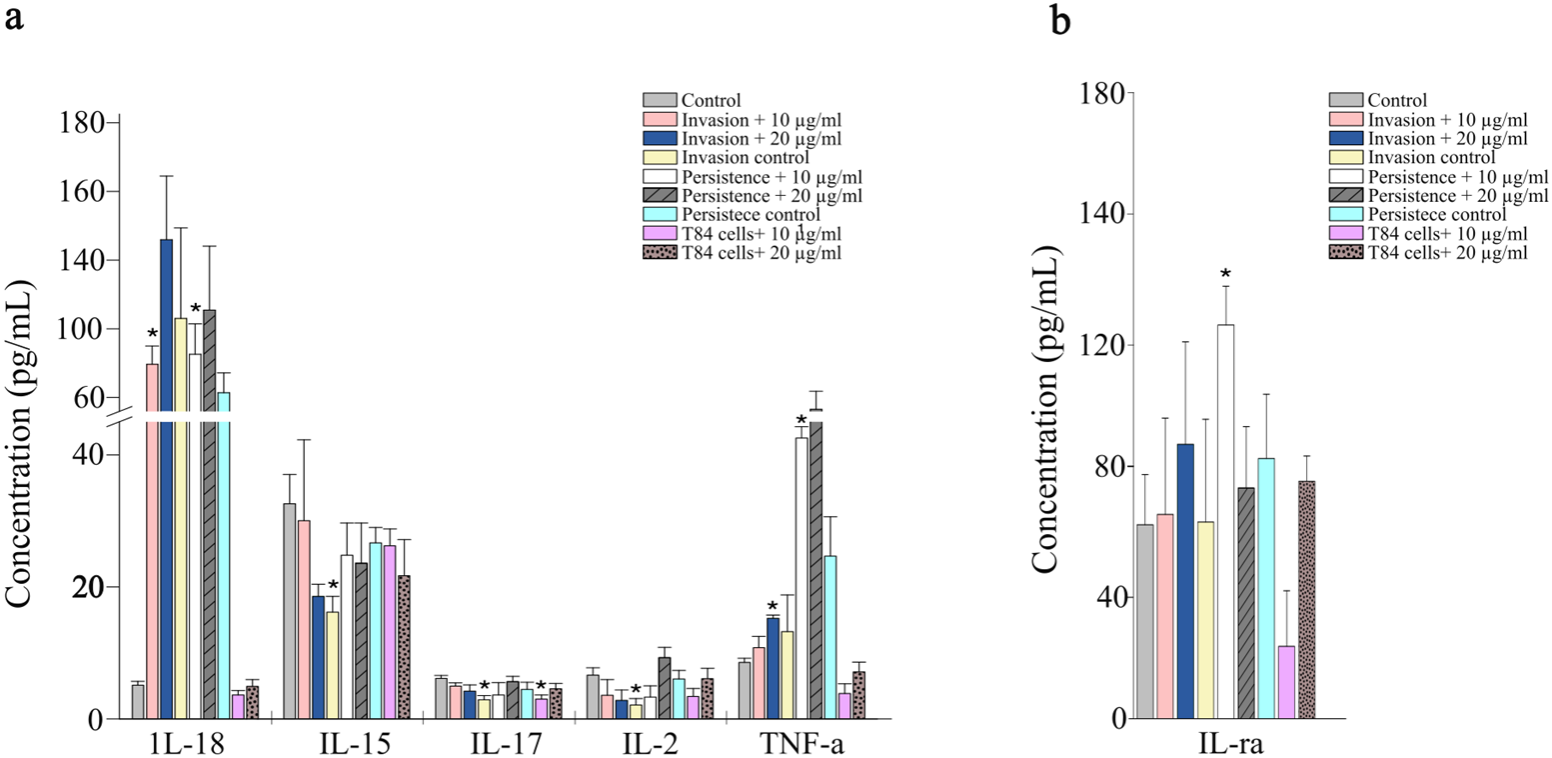
Concentration of pro- and anti-inflammatory cytokines in T84 epithelial cells following AgNPs exposure and bacterial infection. Cytokine concentrations in the supernatants of T84 cells pretreated with 10 µg/mL or 20 µg/mL of 10 nm AgNPs and exposed to *S. enterica* under invasion or persistence conditions. **(a)** Pro-inflammatory cytokines and **(b)** Anti-inflammatory cytokine. Bars represent mean ± SE from biological triplicates. Asterisks indicate statistically significant differences compared to uninfected and AgNPs untreated control (p < 0.05).

T84 cells were treated with 10 nm AgNPs at either 10 or 20 µg/mL, alone or in combination with *Salmonella enterica* infection. Untreated T84 cells served as reference controls and statistical significance was assessed using unpaired Student’s t-tests (p < 0.05). Pretreatment with 10 µg/mL AgNPs in the presence of bacterial infection showed the most notable changes in cytokine secretion, particularly among pro-inflammatory cytokines (**Figure 3a**). IL-18 (Interleukin-18) levels were significantly increased during both invasion and persistence conditions in the presence of 10 µg/mL AgNPs, reaching 79.4 and 85.2 pg/mL, respectively, compared to 5.1 pg/mL in untreated controls (**Figure 3a**). No significant changes were observed in IL-18 levels with AgNPs treatment alone. However, IL-15 significantly decreased during bacterial invasion, but the change was not statistically significant with AgNPs treatment. IL-17 levels were significantly reduced following 10 µg/mL AgNPs treatment alone (3 pg/mL) and during bacterial invasion without AgNPs (2.9 pg/mL), relative to 6.13 pg/mL in the control. In contrast to IL-15, the IL-2 exhibited a statistically significant decrease during bacterial invasion, dropping to 2.11 pg/mL from a control value of 6.65 pg/mL. However, AgNPs treatment alone did not significantly alter IL-2 levels. TNF-α levels increased significantly during bacterial persistence in the presence of 10 µg/mL AgNPs, reaching 42.53 pg/mL, compared to only 8.6 pg/mL in untreated controls. A moderate but significant increase was also observed during invasion with 20 µg/mL AgNPs (15.24 pg/mL). IL-1ra (Interleukin-1 receptor antagonist), an anti-inflammatory cytokine, showed no significant change in most conditions but was significantly increased from 68.4 pg/mL in control cells to 138.9 pg/mL during bacterial persistence in the presence of 10 µg/mL AgNPs (**Figure 3b**).

**Figure 4:**
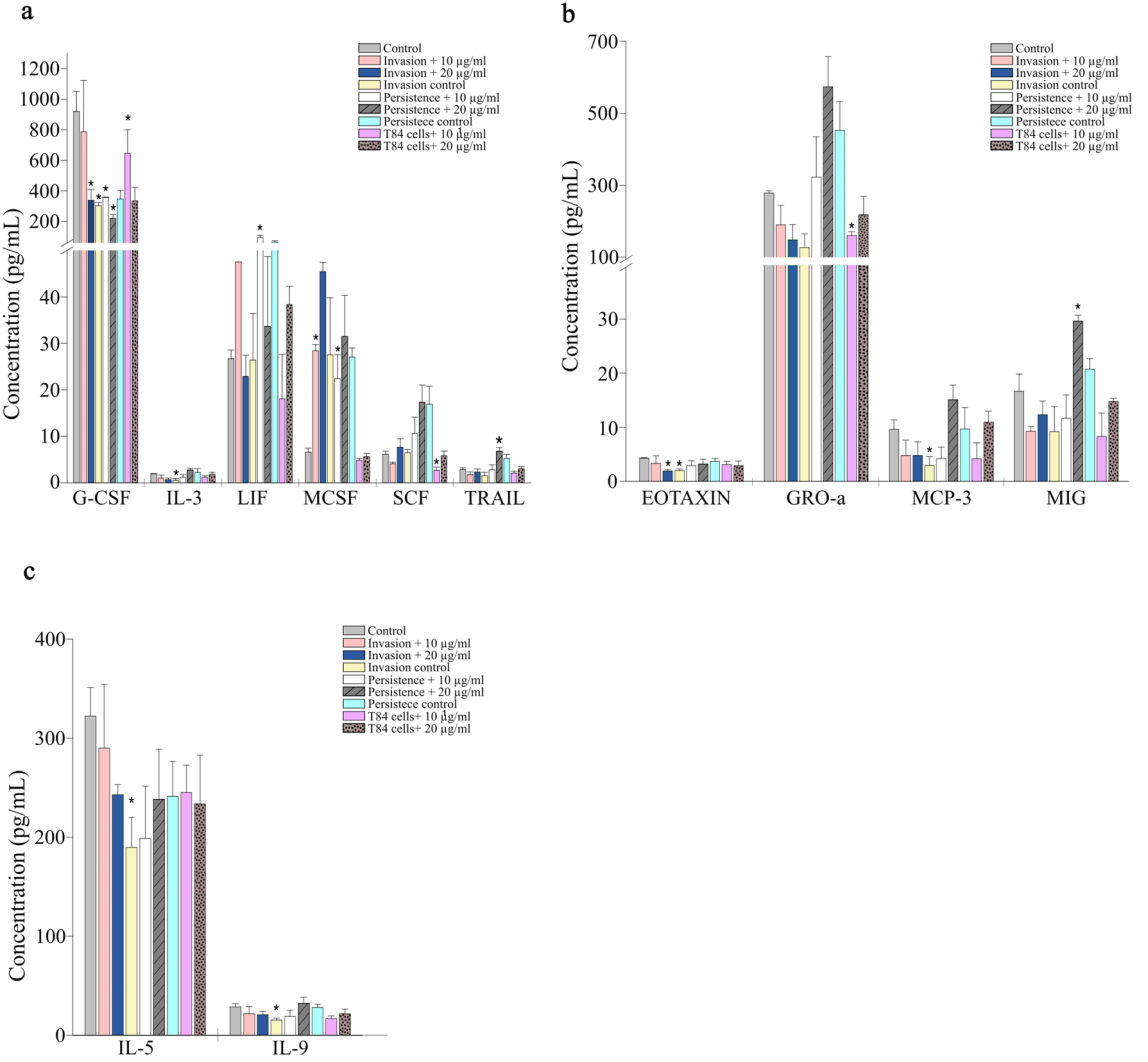
Concentration of growth factors, chemokines, and Th2 cytokines in T84 epithelial cells following AgNPs exposure and bacterial infection. T84 cells were treated with 10 nm AgNPs at 10 µg/mL or 20 µg/mL, either alone or in combination with *S. enterica* invasion or persistence. Invasion control consisted of infected cells that were not pretreated with AgNPs but treated with gentamicin for 1 hour, while persistence control consisted of infected cells not pretreated with AgNPs but treated with gentamicin for 24 hours. **(a)** Growth factors, **(b)** chemokines, and **(c)** Th2 cytokines. Bars represent mean ± SE from biological triplicates. Asterisks indicate statistically significant differences compared to control (p < 0.05).

Several growth factors involved in hematopoiesis and epithelial repair were also affected by AgNPs exposure (**Figure 4a**). G-CSF, granulocyte colony-stimulating factor, was significantly reduced from 920 pg/mL in control cells to 647 pg/mL in cells treated with 10 µg/mL AgNPs, and further reduced during bacterial invasion to 304 pg/mL and to 339 pg/mL when 10 µg/mL AgNPs was added. The most noticeable reduction occurred under bacterial persistence with 20 µg/mL AgNPs, reaching 221 pg/mL. In contrast, LIF (Leukemia Inhibitory Factor) did not show significant change in most treatments but increased significantly from 26.7 to 96 pg/mL during bacterial persistence in the presence of 10 µg/mL AgNPs. Similarly, TRAIL (TNF-related apoptosis-inducing ligand) levels slightly increased from 5.3 pg/mL to 6.8 pg/mL during bacterial persistence in the presence of 20 µg/mL AgNPs. Additional growth factors such as SCF (Stem Cell Factor) was significantly reduced in T84 cells treated with 10 µg/mL AgNPs alone, but not during infection. IL-3 showed a significant decrease under bacterial invasion alone, while M-CSF (Macrophage Colony-Stimulating Factor) concentrations increased significantly during invasion and persistence when AgNPs was present at 10 µg/mL.

Several chemokines involved in immune cell recruitment showed altered secretion profiles in response to AgNPs exposure and bacterial infection (**Figure 4b**). Eotaxin levels were only significantly decreased during bacterial invasion, both with and without the presence of 10 µg/mL AgNPs. In contrast, GRO-a, a neutrophil chemoattractant, increased during bacterial persistence regardless of AgNPs presence, though these changes were not statistically significant. However, it was significantly reduced from 279 pg/mL in control cells to 160 pg/mL following 10 µg/mL AgNPs treatment. MCP-3 (Monocyte-Chemotactic Protein 3) showed a more distinct response under invasion-only conditions, decreasing significantly from 9.6 pg/mL to 3.0 pg/mL in the absence of AgNPs. However, this decrease was not as noticeable in the presence of AgNPs pretreatment. Finally, MIG (Monokine Induced by Interferon-gamma) concentrations remained largely unchanged across treatments, except for a significant increase from 12.3 pg/mL to 29.6 pg/mL during bacterial persistence in the presence of 20 µg/mL AgNPs.

Th2-associated cytokines also responded differently to AgNPs exposure and infection (**Figure 4c**). For instance, IL-5, a Th2 cytokine involved in B-cells activation and eosinophil recruitment, was notably reduced from 322 pg/mL in untreated T84 cells to 190 pg/mL during bacterial invasion and remained reduced across all infection conditions. However, treatment with AgNPs alone (at either 10 or 20 µg/mL) did not result in significant changes in IL-5 levels. IL-9, another Th2-associated cytokine involved in mucosal immunity, decreased during bacterial invasion alone, with levels dropping from 28.7 pg/mL in controls to 15.3 pg/mL.

## 4 Discussion

Silver nanoparticles are widely used for their antimicrobial activity and have been proposed as carriers for drug delivery; however, their interactions with host cells and potential to cause numerous unintended biological effects requires further investigation (Gomes et al., 2021; Mitchell et al., 2021; Gressler et al., 2025). While AgNPs is known to trigger protective anti-inflammatory responses, multiple studies have demonstrated their harmful effect such as oxidative damage, inflammation, and cytotoxicity (McCracken et al., 2015; Liao et al., 2019; Nosrati et al., 2021; Olugbodi et al., 2023). The size, shape, surface coating, and colloidal stability are critical determinants of nanoparticle–host interactions. These aspects have been systematically examined in our previous studies, which identified specific AgNP characteristics associated with altered host responses (Williams et al., 2015; Williams et al., 2016; Orr et al., 2019; Gokulan et al., 2020). Accordingly, the current study was intentionally focused on AgNPs with those defined properties to assess whether they also influence intestinal barrier integrity and susceptibility to infection. Such contradicting responses highlight the importance of evaluating the biological effect of AgNPs exposure under physiologically relevant conditions especially during bacterial infection. In this study, we examined the effect of low concentration of 10 nm AgNPs on the epithelial barrier integrity, immune response, and cell’s susceptibility to *Salmonella enterica* serovar Heidelberg infection, focusing on early invasion and long-term intracellular persistence. This particular strain is a clinically significant non-typhoidal *Salmonella* serovar frequently associated with poultry products and foodborne outbreaks and is notable for its increased propensity for invasive disease and multidrug resistance compared with many other serovars (Folster et al., 2012; Foley et al., 2013; Zhao et al., 2025). Its virulence is mediated by Salmonella pathogenicity islands (SPI-1 and SPI-2), which encode type III secretion systems required for epithelial invasion and intracellular survival (Zhao et al., 2025).

Our results indicated that pre-exposure to 10 nm AgNPs at 10 µg/mL significantly increased the cell susceptibility to bacterial infection. Rather than offering protection, a 24-hour pretreatment with 10 nm AgNPs (especially at 10 µg/mL) significantly increased bacterial invasion and intracellular persistence compared to untreated cells. Interestingly, AgNPs pretreatment didn’t impact the bacterial adhesion to the epithelial cells, but instead facilitated invasion and intracellular survival, even in the presence of gentamicin. Exposure to AgNPs has been shown to reduce mucus secretion and downregulate of mucin gene expression (e.g., MUC1, MUC2) (Georgantzopoulou et al., 2016; Williams et al., 2016; Bredeck et al., 2021). Since mucus is important in trapping bacteria at the cell surface (Bakshani et al., 2018; Sheng and Hasnain, 2022), a reduction in mucus production could result in fewer bacteria adhering to the cells. This explains the observed non-significant change in bacterial adhesion in our experiment (**Figure 1a**). On the other hand, the increased bacterial invasion and persistence could be due to the epithelial damage and increased permeability caused by pretreatment with 10 nm AgNPs as previously reported in earlier studies (Williams et al., 2015; Williams et al., 2016). Such effect was more noticeable at 10 µg/mL than 20 µg/mL, suggesting that lower AgNPs concentrations compromises epithelial barrier integrity rendering the cells to be more susceptible to bacterial infection. At higher concentration (20 µg/mL), AgNPs could have probably aggregated and were not available at threshold level to interact with cells and cause significant changes (Gokulan et al., 2020; Kampfer et al., 2020). Compromised epithelial integrity has been largely associated with increased invasion by multiple enteric pathogens such as *Salmonella*, *E. coli*, *Pseudomonas aeruginosa* and *Listeria monocytogenes* (Vancamelbeke and Vermeire, 2017; Zheng et al., 2021; Dmytriv et al., 2024).

*S. enterica* serovar Heidelberg strain 146 is highly virulent and invasive as it carries IncA/C and VirD4/B4 plasmids that are involved in multidrug resistance and host manipulation respectively (Tsuji et al., 2009; Gokulan et al., 2013; Zhao et al., 2025). Furthermore, it encodes for SPI-1 and SPI-2 effectors, which facilitate invasion and intracellular survival (Srikanth et al., 2011; Zhao et al., 2025). It also carries silver resistance genes (*silA, silE, silS*, and *silP*), enabling it to survive in the presence of AgNPs. Intestinal barrier damages caused by AgNPs exposure may create a permissive environment for *S. enterica* and weaken intracellular defense, leading to enhanced pathogen survival and replication (Zheng et al., 2021). Moreover, exposure to sublethal concentrations of AgNPs is known to influence bacterial gene expression, promote biofilm remodeling, and upregulate virulence mechanisms (Yang and Alvarez, 2015; Grun et al., 2016). Therefore, AgNPs exposure may not only impair host defense but also create conditions that enhance pathogen virulence.

The effect of AgNPs on intestinal barrier integrity was further supported by the altered expression of genes regulating epithelial junctions. Our results showed that pretreatment with lower concentration of AgNPs (10 µg/mL) resulted in more significant differential regulation of intestinal permeability related genes compared to the higher concentration of 20 µg/mL across all experimental conditions (invasion, persistence, and AgNPs alone) (**Figure 2**). Tight junction genes encoding claudins which regulate paracellular permeability and maintain epithelial barrier integrity (Findley and Koval, 2009; Gunzel and Yu, 2013) (e.g., *CLDN4, CLDN12*, and *CLDN15*) were significantly upregulated in the 10 µg/mL AgNPs + invasion and persistence groups. Whereas *CLDN2*, a pore-forming claudin that increases paracellular permeability, was downregulated. These changes suggested a compensatory response to reinforce barrier integrity in response to the stress caused by AgNPs and bacterial infection (Orr et al., 2019; Gokulan et al., 2020). Similarly, tight junction-associated scaffolding and adhesion molecules such as *ICAM1* and *TJP2*, which contribute to tight junction stability and immune signaling (Mochida et al., 2010; Duong et al., 2020), were upregulated during exposure to 10 µg/mL AgNPs and bacterial invasion and persistence. This likely indicates a wound-healing or compensatory response to both AgNPs-induced oxidative stress and bacterial effectors known to disrupt tight junctions (Tafazoli et al., 2003; Gokulan et al., 2013). Also, the upregulation of focal adhesion integrins *ITGA1* and *ITGA3* at the lower AgNPs concentration either alone or during bacterial invasion and persistence, could be a compensatory mechanism in order to stabilize cell-matrix interactions in response to barrier disruption. Focal adhesions are essential for maintaining epithelial polarity and supporting wound healing, especially under oxidative or inflammatory stress (Velasco-Velazquez et al., 2012; Li et al., 2018). This response was associated with the upregulation of the gap junction gene *GJB3*, which facilitates intercellular communication. Increased expression of *GJB3* during stress caused by AgNPs and bacteria is believed to be a cellular strategy to promote communication and barrier repair processes as previously documented during tissue injury and epithelial healing (Mao et al., 2010; Iwashita et al., 2011; Regis et al., 2020).

Among desmosomal and hemidesmosomal genes, *PLEC*, a cytoskeletal crosslinker critical for membrane integrity, showed the highest upregulation, accompanied by increases in *DSP*, *DSG4*, and *DSC3* following 10 µg/mL AgNPs exposure, especially in the presence of bacteria. The upregulation of these genes suggested an attempt to restore barrier function and structural integrity, however the concurrent increase in bacterial invasion and persistence indicated that these compensatory responses were insufficient to overcome AgNPs-induced permeability.

The observed impact of the lower AgNPs concentration on genes associated with intestinal barrier function has been previously reported in both *in vivo* and ex vivo studies, where smaller-sized AgNPs at low concentrations modulated barrier-associated genes (Lim et al., 2012; Williams et al., 2016; Fehaid and Taniguchi, 2019). In contrast, higher concentrations often resulted in minimal transcriptional changes due to increased nanoparticle aggregation, which reduce their ability to interact then penetrate through the intestinal epithelial cells (Gokulan et al., 2020; Kampfer et al., 2020).

Along with barrier integrity changes, AgNPs exposure in the presence of bacterial infection has altered the mucosal immune response of the epithelial cells. Again, treatment with lower AgNPs concentration (10 µg/mL) in combination with bacterial infection showed the most significant changes in cytokine secretion, highlighting concentration-dependent induced mucosal immune response (**Figures 3**, **4**). The changes in cytokines observed with lower AgNPs concentrations has been previously observed (Lim et al., 2012; Williams et al., 2016; Fehaid and Taniguchi, 2019). Pre-exposure to 10 µg/mL AgNPs followed by bacterial infection has resulted in increasing the concentration of key pro-inflammatory cytokines including IL-18 and TNF-α. The increase of IL-18, which is essential for mucosal immunity and inflammasome activity response (Hanamsagar et al., 2011; Ihim et al., 2022), during both bacterial invasion and persistence suggests that AgNPs exposure may increase inflammasome activation in the presence of bacterial infection, and potentially exacerbating epithelial inflammation. Similarly, the concentration of TNF-α which promotes degradation of tight junction proteins and compromises barrier integrity, was significantly elevated during bacterial persistence in the presence of AgNPs (Droessler et al., 2021).

The concurrent upregulation of tight junction genes in our study may therefore represent a compensatory epithelial response to restore barrier function in response to any TNF-α–driven disruption. However, the increased levels of pro-inflammatory cytokines as a result of AgNPs exposure could contribute to the observed increased bacterial invasion and persistence (**Figure 1a**). On the other hand, the anti-inflammatory cytokine IL-1ra, which inhibits IL-1 signaling and reduce inflammation (Powers et al., 2020) remained unchanged across all experimental conditions except during bacterial persistence in the presence of 10 µg/mL AgNPs. This suggested a compensatory response to reduce any tissue damage caused by increased TNF-α and IL-18, however, the elevation in IL-1ra was relatively less compared to the marked increase in pro-inflammatory cytokines. This imbalanced response could facilitate epithelial damage and barrier disruption.

Moreover, growth factors involved in epithelial repair, including G-CSF, LIF and M-CSF, were also changed after AgNPs exposure. The concentrations of G-CSF, which is essential for epithelial regeneration (Park et al., 2022), was reduced as a result of AgNPs exposure during both bacterial invasion and persistence. Accordingly, epithelial restitution might be impaired, and the immune response gets delayed, resulting in increased barrier disruption and susceptibility to microbial invasion. In contrast, the increased levels of LIF and M-CSF under the same conditions suggests a compensatory mechanism in response to the damage caused by exposure to the nanoparticles or bacterial infection. LIF enhances epithelial cell survival and mucosal healing (Wang et al., 2020), and M-CSF plays an important role in mucosal tissue remodeling (Hirata et al., 2010; Yi et al., 2024). However, continuous M-CSF increase can lead to chronic inflammation and epithelial damage (Klebl et al., 2001).

The chemokines MCP-3 and eotaxin, which are responsible for recruitment of monocyte and eosinophil, respectively, were significantly decreased during bacterial infection, particularly without AgNPs. However, GRO-α, a neutrophil chemoattractant, was lowered by 10 µg/mL AgNPs treatment alone but increased during bacterial persistence in the presence of 20 µg/mL AgNPs, although this increase was not statistically significant. This observation suggests that AgNPs suppress chemokine-driven immune recruitment, while bacterial infection may restore specific chemotactic signals like GRO- α as a delayed response to the ongoing inflammation. Th2-associated cytokines such as IL-5 and IL-9 were both reduced during bacterial invasion without AgNPs. IL-5 and IL-9 promote eosinophil activation and recruitment (Ogulur et al., 2025), and their reduction may limits eosinophil activity at the epithelial barrier, reducing their protective roles in maintaining mucosal homeostasis especially during infection. Chemokines involved in immune cell recruitment, including MCP-3 and eotaxin, were significantly reduced during bacterial infection, particularly in the absence of AgNPs. This could lead to impaired immune response and compromised host’s defense against. Collectively, the observed changes of chemokine and cytokine concentrations suggest that AgNPs exposure suppresses early immune recruitment and protective signaling, while enhancing the pro-inflammatory pathways. This imbalance may disrupt immune coordination at the epithelial surface, reducing the containment of *Salmonella* and enabling its invasion and intracellular persistence.

In conclusion, our results showed that exposure to 10 nm AgNPs at 10 µg/mL can significantly compromise epithelial barrier integrity and dysregulate immune function, which might facilitate bacterial invasion. Such effect raises more concerns about the unintended consequences of AgNPs usage, despite being widely recognized as antimicrobial agents. These results underscore the potential health risks of chronic AgNP ingestion and highlight the need to evaluate their safety in the context of gastrointestinal health and host–pathogen interactions. Repeated ingestion of AgNPs may impair the host’s ability to contain enteric opportunistic pathogens, potentially increasing the susceptibility to GIT infections and worsening the inflammatory responses. Another important aspect to consider is the interplay between AgNPs exposure and the gut microbiome homeostasis. While our study didn’t assess microbiome composition, previous work has shown that AgNPs exposure can disrupt microbial communities by inhibiting sensitive bacteria while enriching resistant ones (Tlili et al., 2017; Grün et al., 2019). Notably, genes responsible for silver resistance are not widespread among all gut microbes but are commonly found in Enterobacteriaceae (Finley et al., 2015; Hosny et al., 2019). *S. enterica*, which carries these genes, would have a competitive advantage in the presence of AgNPs, outcompeting other gut bacteria leading to a microbial dysbiosis. This demonstrates a dual effect of AgNPs, compromising the epithelial integrity and altering the normal gut microbiome, which enhances susceptibility to enteric pathogens like *Salmonella*. Therefore, there’s a need to be cautious about AgNPs’ potential to inadvertently compromise host defense and increase host susceptibility to infection, especially with prolonged or unregulated exposure. This work also highlights the need to include host-pathogen interaction models in the nanotoxicology assessment studies to fully understand the consequences of chronic or repeated AgNPs exposure on the gut health.

## Data Availability

All data generated in this manuscript will be made available by the authors to any qualified researcher as per the guidelines of US-Food and Drug Administration data sharing policy.
